# Artificial intelligence based diagnosis of sulcus: assesment of videostroboscopy via deep learning

**DOI:** 10.1007/s00405-024-08801-y

**Published:** 2024-07-13

**Authors:** Ömer Tarık Kavak, Şevket Gündüz, Cabir Vural, Necati Enver

**Affiliations:** 1 Department of Otorhinolaryngology, Marmara University Faculty of Medicine, Pendik Training and Research Hospital, Fevzi Çakmak Muhsin Yazıcıoğlu Street, İstanbul, 34899 Turkey; 2 VRLab Academy, 32 Willoughby Rd, Harringay Ladder, London, N8 0JG UK; 3 Marmara University Faculty of Engineering, Electrical and Electronics Engineering, Başıbüyük, RTE Campus, İstanbul, 34854 Turkey

**Keywords:** Sulcus, Deep learning, Laryngoscopic image, Artificial intelligence, Convolutional neural networks

## Abstract

**Purpose:**

To develop a convolutional neural network (CNN)-based model for classifying videostroboscopic images of patients with sulcus, benign vocal fold (VF) lesions, and healthy VFs to improve clinicians’ accuracy in diagnosis during videostroboscopies when evaluating sulcus.

**Materials and methods:**

Videostroboscopies of 433 individuals who were diagnosed with sulcus (91), who were diagnosed with benign VF diseases (i.e., polyp, nodule, papilloma, cyst, or pseudocyst [311]), or who were healthy (33) were analyzed. After extracting 91,159 frames from videostroboscopies, a CNN-based model was created and tested. The healthy and sulcus groups underwent binary classification. In the second phase of the study, benign VF lesions were added to the training set, and multiclassification was executed across all groups. The proposed CNN-based model results were compared with five laryngology experts’ assessments.

**Results:**

In the binary classification phase, the CNN-based model achieved 98% accuracy, 98% recall, 97% precision, and a 97% F1 score for classifying sulcus and healthy VFs. During the multiclassification phase, when evaluated on a subset of frames encompassing all included groups, the CNN-based model demonstrated greater accuracy when compared with that of the five laryngologists (%76 versus 72%, 68%, 72%, 63%, and 72%).

**Conclusion:**

The utilization of a CNN-based model serves as a significant aid in the diagnosis of sulcus, a VF disease that presents notable challenges in the diagnostic process. Further research could be undertaken to assess the practicality of implementing this approach in real-time application in clinical practice.

**Supplementary Information:**

The online version contains supplementary material available at 10.1007/s00405-024-08801-y.

## Introduction

Sulcus is a pathological condition affecting the vocal folds (VF) that is characterized by the presence of a sulcus, which refers to an indentation, groove, or furrow located at the edge of the VF [1]. The formation of the sulcus correlates with a rise in collagen fiber density. This process may occur solely in the superficial layer of the lamina propria along the VF edge; in more severe cases, it extends through the vocal ligament and reaches the thyroarytenoid muscle [2]. The sulcus is distinguished by the presence of spindle-shaped glottal insufficiency during phonation, resulting in heightened air leakage. This condition is further defined by augmented VF stiffness, which manifests as a high-pitched, rough, breathy, and asthenic voice. Patients with sulcus also exhibit compensatory supraglottal hyperactivity. The observed clinical manifestations encompass hoarseness, vocal fatigue, and exertion during vocalization [1].

Though sulcus is categorized as a condition affecting the VFs, diagnosing and distinguishing it from other conditions is difficult due to its subtle manifestations. Unlike anatomical abnormalities (e.g., cysts and polyps), sulcus presents with a less pronounced appearance. Videostroboscopy is the current gold-standard examination for this purpose [3]. It is established by reduced amplitude of the mucosal wave, a bowed or curved aspect to the free edge of the involved VF, glottic incompetence, and compensatory hyperfunction of the ventricular folds in certain cases [1, 2]. Nevertheless, VFs may appear normal during in office laryngoscopy [4]. Difficulties in diagnosing sulcus with the use of videostroboscopy have also been documented by Dailey et al. [5] and Sunter et al. [6]. According to Dailey et al. [5], sulcus is a mucosal condition that is commonly misdiagnosed. In addition, videostroboscopy yields qualitative data, leading to significant interindividual variability, with which untrained clinicians may struggle when attempting to accurately diagnose sulcus [7]. The infrequent incidence of the disease and subsequent lack of familiarity among clinicians, along with the challenges associated with videostroboscopic diagnosis, have led clinicians to explore auxiliary computer-based diagnostic approaches.

Due to the progress in computational capabilities, extensive utilization of deep learning techniques (e.g., convolutional neural networks [CNN]) has contributed to significant advancements in artificial intelligence (AI) models, particularly in the field of image recognition [8]. Videostroboscopy, a popular laryngoscopic procedure executed by otolaryngologists, provides a wealth of images for AI models in machine learning and computer vision. Furthermore, the availability of such a substantial dataset has facilitated the application of AI in the field of otolaryngology, leading to an increase in publications on this subject [9–11]. These studies encompass a wide range of applications, including lesion recognition, vibration analysis, determination of vocal cord movement, identification and characterization of laryngeal structures, image enhancement, and informative frame selection [10].

Despite the presence of various existing studies related to the diagnosis and classification of both benign and malignant VF lesions, no studies have specifically addressed the inclusion of sulcus. In this study, our main objective is to construct a CNN-based model for distinguishing videostroboscopic images to differentiate sulcus from those corresponding to healthy groups and benign VF lesions such as polyps, nodules, papillomas, cysts, and pseudocysts. This aims to improve the clinician-based accuracy of diagnostic assessments of laryngoscopy findings.

## Materials and methods

A retrospective study was conducted in accordance with the regulations of the Declaration of Helsinki after the approval of the tertiary medical faculty Ethics Committee for Clinical Research on October 27, 2023, with number 09.2022.1449. All patient data was anonymized and utilized in a blind manner. Because the study was designed retrospectively, informed consent was not obtained from the patients.

**Fig. 1:**
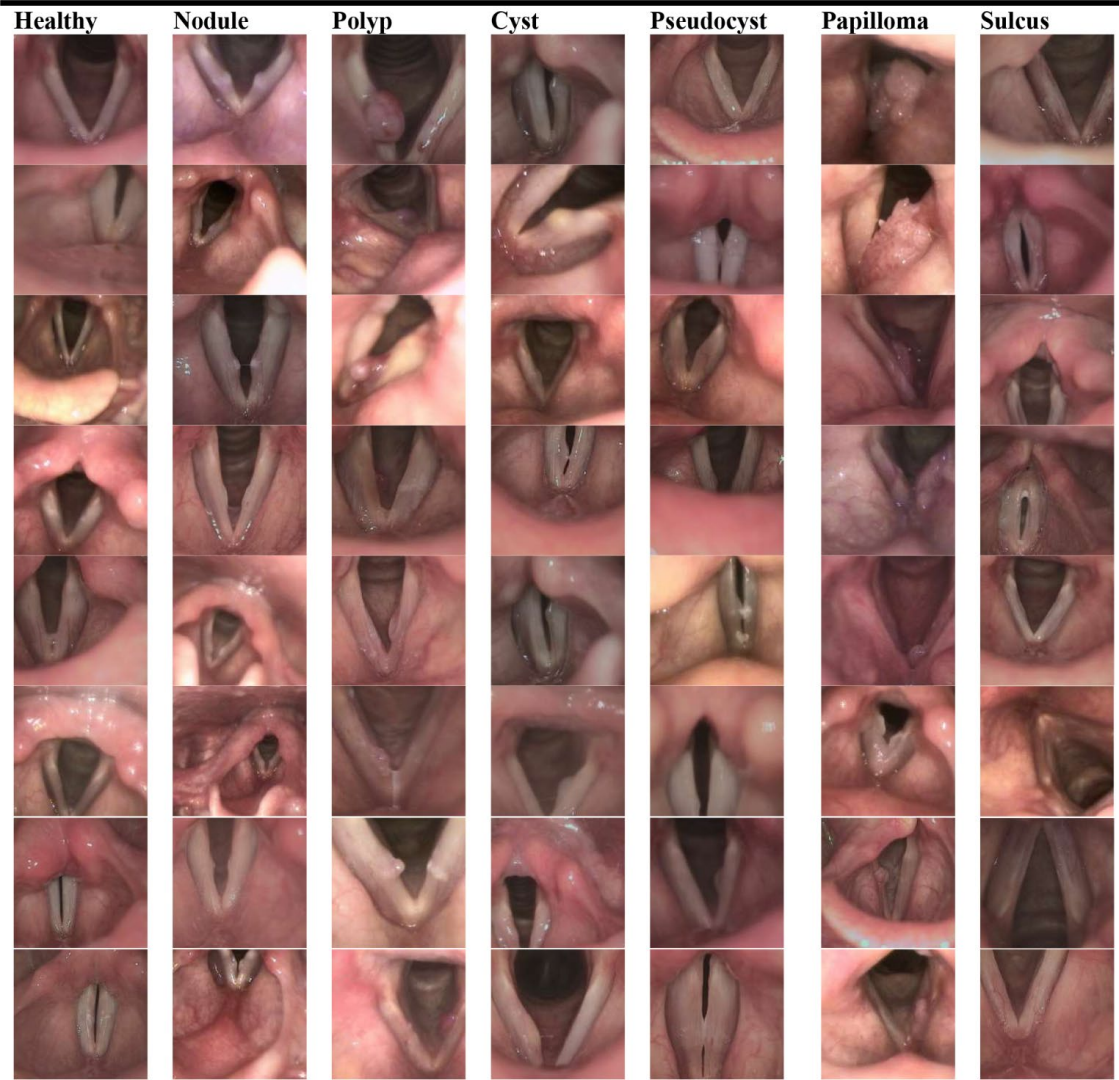
Representative frames from each subset (Healthy, Nodule, Polyp, Cyst, Pseudocyst, Papilloma, Sulcus) in the dataset employed for convolutional neural network (CNN) model development

The requisite frames for training the CNN-based model were obtained from the videostroboscopy database maintained by the Ear, Nose, and Throat Department of a tertiary medical center. Data was collected for the period from 2019 to 2023. Videostroboscopy procedures were performed using a rigid 8 × 180 mm or flexible 3.9 mm laryngoscope from XION Medical Systems. No exclusion criteria were imposed based on age, gender, or race. Videostroboscopies of 433 cohorts belonging to seven categories were retrospectively evaluated: healthy, polyps, nodules, papilloma, cysts, pseudocysts, and sulcus. Videos were recorded at a frame rate of 25 frames per second (fps). The size of each frame is $768\times 576$ pixels. Fellowship-trained laryngologists with at least five years of experience who were involved in the conducted study ensured accurate diagnoses and the incorporation of confirmed pathological diagnosis (N.E). Frames from the relevant records were selected to create a dataset. Specifically, the focus was on VF lesions, excluding frames with underexposure, blurriness, saliva, or specular reflections. The process of dataset preparation yielded a total of 91,159 informative frames. Each individual frame was resized to $451\times 468$ pixels to eliminate peripheral areas and saved in red-green-blue (RGB) color format. Authors in the field of otorhinolaryngology (Ö.T.K, N.E) meticulously examined each frame in the videos and categorized it based on the disease indicators. Table 1 shares the details of the number of participants and frames selected from each study group. Figure 1 provides examples of annotated images.

**Table 1:** Number of patients and representative frames in each study group

Subset label	Name of subset	Number of patients	Number of frames
0	Healthy	33	8889
1	Nodule	99	11,517
2	Papilloma	16	6570
3	Polyp	132	37,359
4	Sulcus	91	13,409
5	Cyst	50	11,779
6	Pseudocyst	12	1636
	Total	433	91,159

The study developed two classifiers. Based on the existing literature, it is evident that the sulcus represents the most commonly undiagnosed benign VF lesion, and it may appear normal during indirect laryngoscopy [1, 5]. A binary classifier was then developed with the goal of distinguishing an image corresponding to a patient diagnosed with sulcus from one corresponding to a healthy subject. In the second phase of the study, a multi-class classifier was developed to classify a given image into one of seven distinct categories. The “Supporting Information” section describes the overall architecture of the CNN-based model.

Selected frames from the original dataset were divided into appropriate sets to train, validate, and test the CNN-based classifiers. Furthermore, survey data collected from the original dataset was used to assess the classification performance of the proposed CNN-based model in comparison to five laryngologists who were not involved in the study. Table 2 provides a summary of the number of frames utilized during the training, validation, and testing stages in both binary and multi-class classification and presents the number of frames collected specifically for survey data.

**Table 2:** Number of frames used in training, validation, testing, and Survey Data for Binary and Multi-class classification models

Binary classification									
Subsets		Training		Validation		Testing			Total
0:Healthy		5575		1394		1743			**8712**
1:Sulcus		5575		1394		1743			**8712**
**Total**		**11,150**		**2788**		**3486**			**17,424**
**Multi-class classification**									
**Subsets**	**Training**		**Validation**		**Testing**		**Survey**	**Total**	
0:Healthy	3147		786		1066		104	**5103**	
1:Nodule	3205		801		992		75	**5073**	
2:Papilloma	3216		804		980		100	**5100**	
3:Polyp	3190		797		1013		75	**5075**	
4:Sulcus	3184		803		984		40	**5011**	
5:Cyst	3217		804		979		75	**5075**	
6:Pseudocyst	1032		258		305		75	**1670**	
**Total**	**20,191**		**5053**		**6319**		**544**	**32,107**	

A preprocessing stage was performed to improve overall efficiency, which involved converting the frames from the RGB color format to grayscale, resulting in a reduction of color channels from three to one. Additionally, the frames were resized to 25% of their original dimensions, and original pixel values ranging from 0 to 255, were normalized to the [0,1] range. As a consequence, the size of the frames was reduced to $112\times 117$ pixels in grayscale format for multi-class classification and to $45\times 46$ pixels in RGB format for binary classification.

The adaptive momentum (ADAM) algorithm was employed to iteratively optimize the model parameters (see Supporting Information, Methods). Supporting Table 1 lists the optimal values of the hyperparameters for binary and multi-class classifiers. Various evaluation metrics (e.g., accuracy, recall, precision, F1-score, macro average F1-score [average of F1 scores calculated in multiclassification], Cohen-Kappa score [CKS], and Matthews correlation coefficient [MCC]) values were employed to assess classifier performance.

## Results

**Fig. 2:**
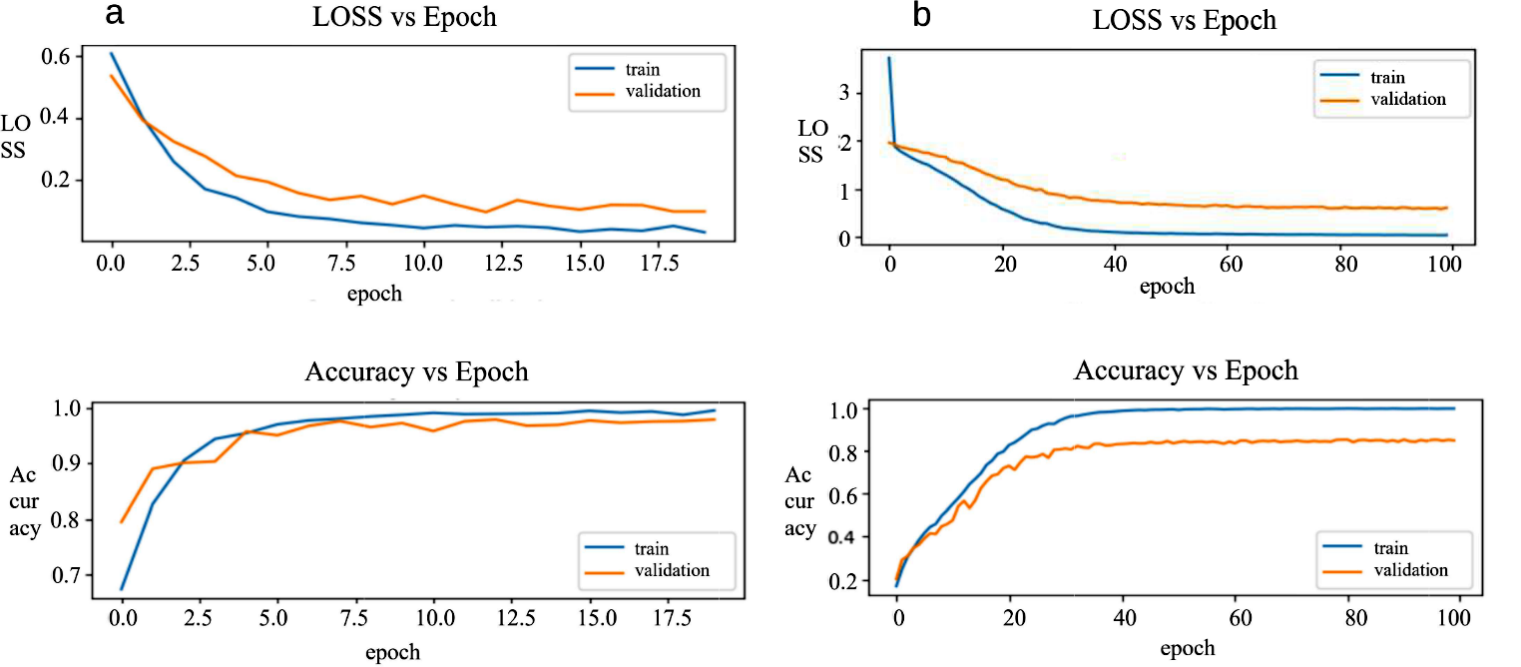
Accuracy and loss curves of the proposed convolutional neural network (CNN) models across epochs in training and validation processes: (**a**) binary classifier, (**b**) multi-class classifier

Figure 2 illustrates the accuracy and loss curves of the classifiers with respect to epoch number during training and validation processes. These curves were achieved by using the optimal hyperparameters in the CNN-based models. The CNN-based model achieved a performance of over 95% in all metrics in the binary classification phase for detecting frames indicating sulcus. Table 3 provides the model’s predictions regarding the classification of frames into respective subsets as a confusion matrix and metrics derived from the confusion matrix.

**Table 3:** The confusion matrix and performance metrics for the convolutional neural network (cnn) - based binary classifier

		Actual Label		Metric	Value
		Positive: Sulcus	Negative: Healthy		
Predicted Label	Positive: Sulcus	TP: **1739**	FP: **37**	Accuracy (%)	98
				Recall (%)	97
	Negative: Healthy	FN: **25**	TN: **1684**	Precision (%)	98
				F1 Score (%)	97
				Loss	0.087

*Abbreviations*: *TP* True Positive; *FP* False Positive; *TN* True Negative; *FN* False Negative

**Fig. 3:**
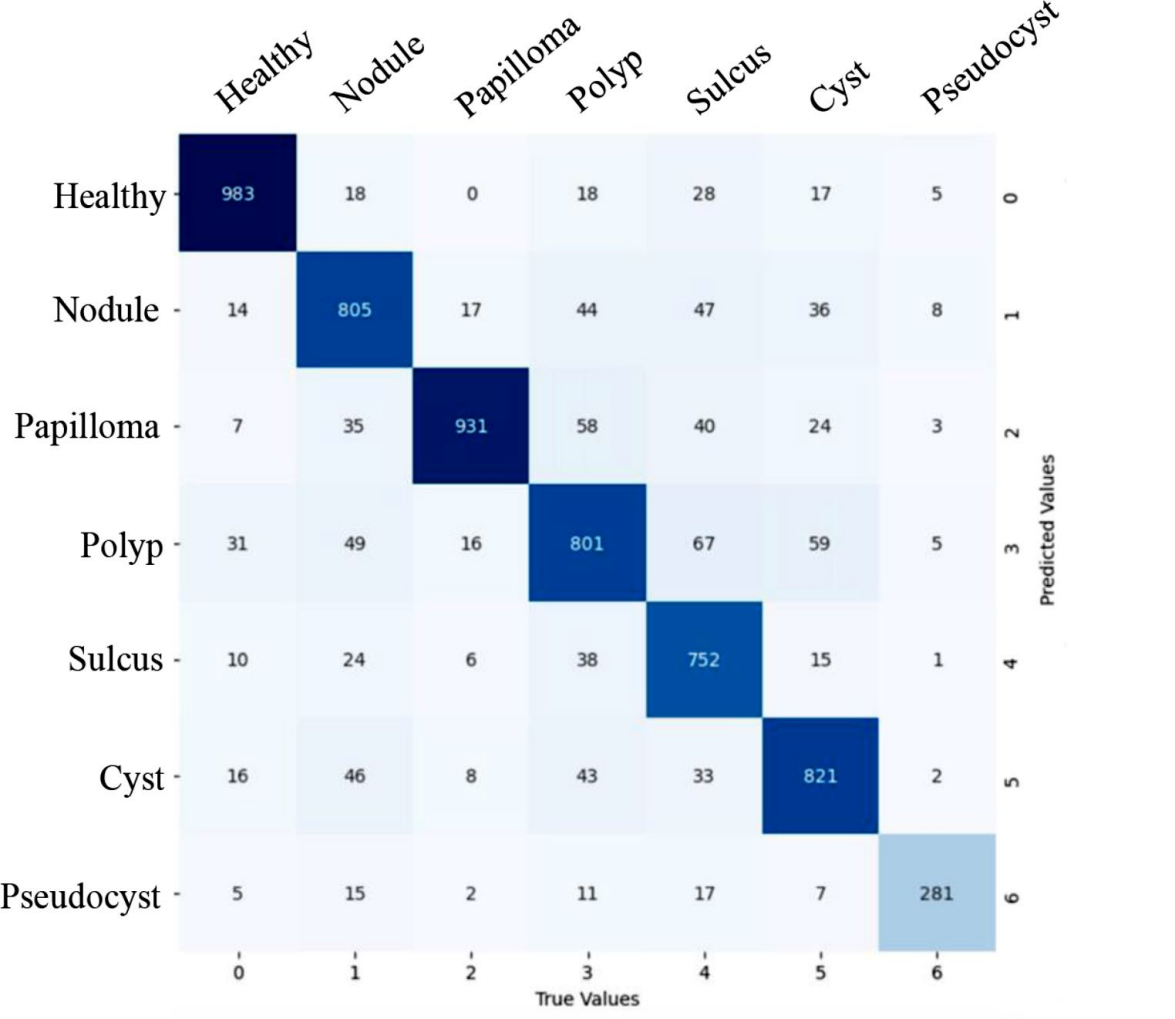
Confusion matrix for the convolutional neural network (CNN)-Based multi-class classifier

The subsequent phase of the study aimed to evaluate the model’s multi-class classification performance in testing data that included 6,319 frames from all included groups. The CNN-based model achieved 85% accuracy in the multi-class case. Table 4; Fig. 3 present the detailed results for other metrics and the confusion matrix. To compare classification performance in survey data that included 544 frames, the confusion matrices and performance metrics of the CNN-based model and laryngologists were reviewed.

**Table 4:** Performance metrics of the convolutional neural network (CNN)-based model and laryngologists in testing and survey data for multi-classification

	Group	Recall	Precision	F1 Score	Accuracy, CKS*, MCC**
CNN in Testing Data (6319 frames)	0:Healthy	92%	92%	92%	Accuracy: 85% CKS: 0.8234 Average MCC: 0.8238
	1:Nodule	81%	83%	82%	
	2:Papilloma	95%	85%	90%	
	3:Polyp	79%	78%	78%	
	4:Sulcus	76%	89%	82%	
	5:Cyst	84%	85%	84%	
	6:Pseudocyst	92%	83%	87%	
CNN in Survey data (544 frames)	0:Healthy	80%	85%	82%	Accuracy:76% CKS: 0.7164 Average MCC: 0.7169
	1:Nodule	76%	69%	72%	
	2:Papilloma	91%	80%	84%	
	3:Polyp	73%	74%	73%	
	4:Sulcus	78%	72%	74%	
	5:Cyst	73%	74%	74%	
	6:Pseudocyst	82%	79%	80%	
Clinician 1 in survey data (544 frames)	0:Healthy	88%	66%	75%	Accuracy:72% CKS: 0.6593 Average MCC : 0.6708
	1:Nodule	68%	84%	75%	
	2:Papilloma	81%	92%	87%	
	3:Polyp	96%	58%	72%	
	4:Sulcus	63%	82%	71%	
	5:Cyst	43%	86%	57%	
	6:Pseudocyst	25%	59%	35%	
Clinician 2 in survey data (544 frames)	0:Healthy	71%	76%	73%	Accuracy:68% CKS: 0.6282 Average MCC :0.6326
	1:Nodule	76%	63%	69%	
	2:Papilloma	80%	98%	88%	
	3:Polyp	88%	72%	79%	
	4:Sulcus	59%	72%	65%	
	5:Cyst	39%	74%	51%	
	6:Pseudocyst	50%	28%	36%	
Clinician 3 in survey data (544 frames)	0:Healthy	88%	68%	77%	Accuracy:72% CKS: 0.6593 Average MCC :0.6707
	1:Nodule	68%	78%	73%	
	2:Papilloma	80%	95%	87%	
	3:Polyp	95%	57%	71%	
	4:Sulcus	64%	83%	72%	
	5:Cyst	44%	87%	58%	
	6:Pseudocyst	25%	62%	36%	
Clinician 4 in survey data (544 frames)	0:Healthy	62%	73%	67%	Accuracy:63% CKS: 0.5623 Average MCC: 0.5721
	1:Nodule	77%	67%	72%	
	2:Papilloma	81%	95%	88%	
	3:Polyp	87%	52%	65%	
	4:Sulcus	55%	72%	62%	
	5:Cyst	37%	70%	49%	
	6:Pseudocyst	5%	22%	8%	
Clinician 5 in survey data (544 frames)	0:Healthy	89%	66%	76%	Accuracy:72% CKS: 0.6594 Average MCC:0.6699
	1:Nodule	67%	82%	74%	
	2:Papilloma	84%	91%	87%	
	3:Polyp	94%	58%	72%	
	4:Sulcus	63%	82%	71%	
	5:Cyst	44%	85%	58%	
	6:Pseudocyst	23%	56%	32%	

*Cohen-Kappa Score (CKS)

** Matthews Correlation Coefficient (MCC)

**Fig. 4:**
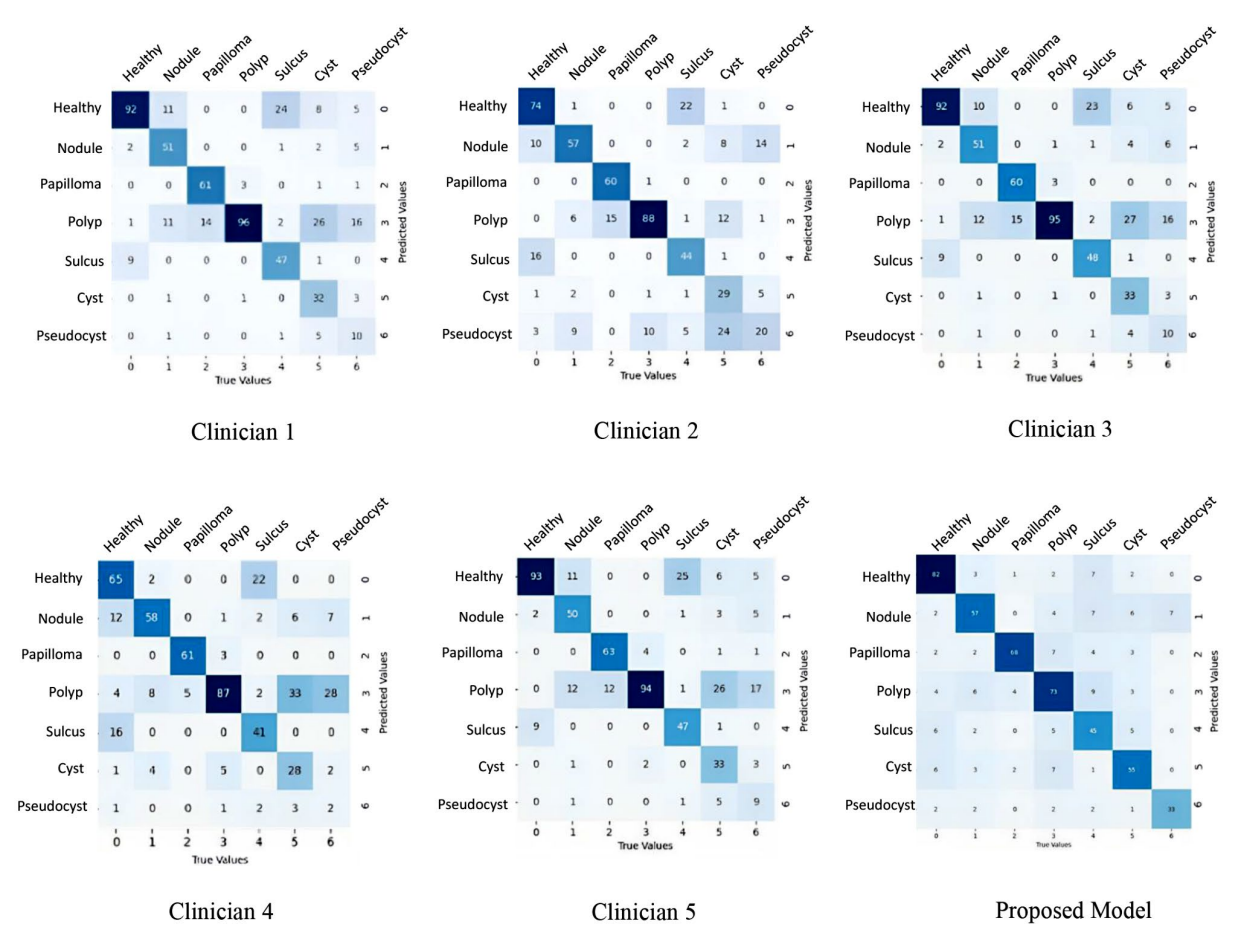
The confusion matrices for all participants, including the proposed model, based on survey data

The CNN-based model showed comparable recall (80% versus 62–89% [average 79.6%]) and notably high precision metrics (85% versus 66–76% [average 69.8%]) in the healthy group in comparison to the performance of laryngologists. In the nodule group, the CNN-based model achieved comparable recall (76% versus 67–77% [average 71.2%]) and precision metrics (69% versus 63–84% [average 74.8%]) to those achieved by laryngologists. In the papilloma group, the CNN-based model had higher recall (91% versus 80–84% [average 81.2%]) performance but lower precision performance (80% versus 91–98% [average 94.2%]) compared to laryngologists. In the polyp group, the CNN-based model demonstrated lower recall (73% versus 87–96% [average 92%]) but higher precision (74% versus 52–72% [average 59.4%]) when compared to laryngologists. In the sulcus group, the CNN-based model showed higher recall (78% versus 55–64% [average 60.8%]) and comparable precision metrics (72% versus 72–83% [average 78.2%]) to those achieved by laryngologists. In the cyst group, the CNN-based model was shown to achieve higher levels of recall (73% versus 37–44% [average 41.4%]) and comparable precision performance (74% versus 70–87% [average 80.4%]) to those achieved by laryngologists. Lastly, for the pseudocyst category, the CNN-based model showed higher levels of recall (82% versus 5–50% [average 25.6%]) and precision metrics (79% versus 22–62% [average 45.4%]) in comparison to the performance of laryngologists. Furthermore, the CNN-based model outperformed laryngologists, as it achieved higher scores across various metrics such as accuracy, CKS, macro average F1-score, and MCC. These results highlight the robust performance of the CNN-based model in comparison to expert laryngologists in the multi-class classification task. Table 4 displays accuracy, recall, precision, CKS, macro average F1-score, and average MCC values, and Fig. 4 depicts the relevant confusion matrices. As a final remark, the CNN-based model not only demonstrated superior accuracy in classification but also achieved much faster classification times compared to the laryngologists (CNN: milliseconds versus C1: 32 min, C2: 42 min, C3: 30 min, C4: 36 min, and C5: 35 min).

## Discussion

Sulcus refers to a groove located at the free edge of the VF, which is typically linked to a reduced vibration pattern [1]. In conjunction with clinical symptoms, videostroboscopy serves as a diagnostic modality for sulcus. Nevertheless, the process of diagnosis can present challenges, as evidenced by the existing literature [5, 6]. It is subjective and highly dependent on the examiner’s expertise. Additionally, not all physicians have sufficient training, experience, or equipment to fully visualize the larynx for a diagnosis of VF disease. These difficulties have necessitated the development of supplementary computer-based systems that incorporate AI to aid clinicians in the diagnostic process.

In our study, two CNN-based classifiers were developed for the purpose of distinguishing images of patients diagnosed with sulcus from those with other VF diseases and from healthy individuals. The proposed models demonstrated promising performance metrics. Specifically, the binary classifier reached an accuracy of 98% and an F1 value of 97%. The multi-class classifier achieved 85% accuracy, which is comparable to clinicians in distinguishing between sulcus, healthy individuals, and benign VF diseases. Our study’s findings indicate that the utilization of CNN-based models holds promise for enhancing clinical laryngoscopy assessments and has the potential to contribute to the development of a contemporary automated system for diagnosing challenging conditions such as sulcus.

Previous studies have investigated AI models in various applications within the field of laryngology (i.e., glottal area segmentation, VF vibration analysis, movement determination, and lesion recognition) [10]. In the context of VF lesion recognition and classification, most studies used traditional machine learning methods such as support vector machines (SVM) and k-nearest neighbors [12–20]. However, a limited number of studies employed deep learning algorithms (specifically CNN), which exhibit significant computational capabilities when using larger datasets, particularly in the field of lesion recognition and classification [21–26]. In addition, assessment and classification of the sulcus with CNN-based models were not conducted in any studies. To our knowledge, only a single study by Turkmen et al. classified sulcus along with a set of VF diseases in the present literature [19]. Turkmen et al. used a region-growing and vessel-linking-based segmentation algorithm to segment blood vessels. They used features extracted from the blood vessels as input into SVM, random forest, and k-nearest neighbors classification algorithms to classify images into healthy, polyp, nodule, sulcus, and laryngitis groups. Then, they assessed the performance of their method using laryngeal pictures from 70 patients, and found that the sensitivities for the healthy, polyp, nodule, laryngitis, and sulcus classes were 86%, 94%, 80%, 73%, and 76%, respectively. Nevertheless, Turkmen et al. employed a binary decision tree model that integrates human expertise and machine learning techniques to effectively classify VF. To the best of our knowledge, our study is the first to assess sulcus using a CNN-based model. In contrast to Turkmen et al., our classification process did not involve human intervention. On the contrary, we solely utilized an AI-based model. Human intervention was limited to the preparation of the whole dataset for the purpose of training and testing the proposed classifiers and the formation of the survey data.

Conducting comparative research between AI models and clinicians is of the utmost importance to validate and promote widespread utilization of AI applications as diagnostic tools [27]. Ren et al. [24] and Xiong et al. [26] conducted research for the purpose of AI-based classification of normal, precancerous, and cancerous laryngeal lesions, and the studies involved a comparison of their respective findings with those of clinicians. The researchers demonstrated in their studies [24, 26] that their CNN-based model exhibited higher accuracy rates in classifying lesions compared to human experts. In our study, we compared the performance metrics of the CNN-based model and those of clinicians. Despite imposing no constraint in the frame inclusion process to form the datasets, the F1 score of the CNN-based classifier surpassed the average F1 scores achieved by five laryngologists across various groups, including healthy, polyp, sulcus, cyst, and pseudocyst. However, in the case of the nodule and papilloma groups, the F1 score of the CNN-based classifier was observed to be inferior. The clinicians included in this study were laryngologists with at least five years of experience in the field of laryngology. In this context, we obtained noteworthy results by comparing the laryngologist model to the AI model. Nevertheless, it is imperative to acknowledge that the diagnostic data presented to clinicians consisted of videotroboscopy frames and that the accuracy of clinicians in classifying lesions would have been enhanced by the demonstration of videostroboscopy.

In our study, the dataset was created by extracting frames from videostroboscopies recorded at 25 fps. Considering the fast inference ability of the proposed models, developing advanced AI models to make real-time assessments during laryngoscopies should be a subject of future research. To our knowledge, a single report in the laryngology field by Azam et al. [25] investigates the application of a CNN model for the purpose of real-time assessment of laryngeal squamous cell cancer during both white light and narrow-band imaging video laryngoscopies. Given this gap in the existing literature, the next phase of our research will focus on developing advanced AI models in real-time videostroboscopy settings. Through the implementation of this method, the potential for improving clinicians’ accuracy in diagnosis during laryngoscopies may be boosted, particularly when evaluating challenging lesions such as sulcus.

This study has certain limitations. The inclusion of images obtained from videostroboscopy as data for the AI algorithm, as opposed to the assessment of videostroboscopy itself, results in the elimination of many videostroboscopy findings that are routinely assessed in the diagnostic process of sulcus by clinicians. In our study, evaluating individual frames rather than entire videos may restrict clinicians’ capacity to accurately assess sulcus. Another limitation of our study pertains to the inclusion of patients who did not undergo routine biopsy or surgery. The criteria for inclusion in the dataset relied primarily on the evaluation of videostroboscopies by a fellowship-trained laryngologist (N.E.) with at least five years of experience rather than on pathologically confirmed disease or during suspension microlaryngoscopy examination (10 pseudocyst, 90 nodule cases that were not pathologically confirmed, and 20 sulcus patients did not undergo suspension microlaryngoscopy). Nonetheless, due to its unique visual characteristics and unanimous validation by all specialists, the precision of the dataset per se is ensured.

## Conclusion

This study proposes a CNN-based approach that facilitates the diagnosis of sulcus using videotroboscopy frames. The CNN-based algorithm our research describes has the potential to be trained using diverse datasets pertaining to various VF diseases. Improved deep learning models trained on larger datasets are likely to increase the accuracy and applicability of classifiers in diagnosing VF lesions. Additionally, our dataset can be utilized in multicenter collaboration for training upcoming deep learning models in the field of interest.

## Electronic supplementary material

Below is the link to the electronic supplementary material.

Supplementary Material 1

Supplementary Material 2
