# Supplementary material for: Artificial intelligence based diagnosis of sulcus: assesment of videostroboscopy via deep learning
Source: Eur Arch Otorhinolaryngol. 2024 Jul 13;281(11):6083–91. doi: 10.1007/s00405-024-08801-y (PMC11512876; doi:10.1007/s00405-024-08801-y)
Supplement: Supplementary file 1 — Supplementary Material 1 [file 405_2024_8801_MOESM1_ESM.docx]

**ARTIFICIAL INTELLIGENCE BASED DIAGNOSIS OF SULCUS: ASSESMENT OF VIDEOSTROBOSCOPY VIA DEEP LEARNING**

Ömer Tarık Kavak^1^, Şevket Gündüz^2^, Cabir Vural^3^, Necati Enver^1*^

1:Marmara University Faculty of Medicine, Pendik Training and Research Hospital, Department of Otorhinolaryngology, İstanbul, Turkey

2:VRLab Academy

3:Marmara University Faculty of Engineering, Electrical and Electronics Engineering, İstanbul, Turkey

^*^Corresponding Author: Ömer Tarık Kavak M.D.

Adress: Fevzi Çakmak, Muhsin Yazıcıoğlu Street, 34899, İstanbul, Turkey

[omrkavak11@gmail.com](mailto:omrkavak11@gmail.com) , orcid ID: 0000-0003-2603-3866

+90 531 915 01 31

Author: Şevket Gündüz, Assistant Professor of Physics

Adress: 32 Willoughby Rd, Harringay Ladder, London N8 0JG, United Kingdom

[sevketgunduz@gmail.com](mailto:sevketgunduz@gmail.com.r) , orcid ID: 0000-0002-1847-949X

+90 530 883 49 83

Author: Cabir Vural, Professor of Electrical and Electronics Engineering

Adress: Başıbüyük, RTE Campus, 34854, İstanbul, Turkey

[cabir.vural@marmara.edu.tr](mailto:cabir.vural@marmara.edu.tr) , orcid ID: 0000-0003-2603-3866

+90 555 733 39 87

Author: Necati Enver M.D., Associate Professor of Otorhinolaryngology, FEBORL-HNS

Adress: Fevzi Çakmak, Muhsin Yazıcıoğlu Street, 34899, İstanbul, Turkey

[necatienver@gmail.com](mailto:necatienver@gmail.com) , orcid ID: 0000-0002-3161-8810

+90 532 338 20 88

| **Supporting Table 1**  Hyperparameters of the Convolutional Neural Network (CNN)-Based Model for Multi-Class and Binary Classification | | |
| --- | --- | --- |
| **Layers** | **Parameters (multi-classification)** | **Parameters (binary classification)** |
| Input layer | Image dimension = 112x117x1x1 | Image dimension = 45x46x3x1 |
| Conv 2D | Number of Filters = 16,  Filter Size = (5x5),  Stride = (1,1),  Padding = Valid,  Regularizer = L2 (0.002),  Activation Function = ReLU | Number of Filters = 10,  Filter Size = (5x5),  Stride = (1,1),  Padding = Valid,  Regularizer = L2 (0.002),  Activation Function = ReLU |
| Dropout | Dropout ratio = 0.5 | Dropout ratio = 0.5 |
| Max-pooling | pool size = (2,2) | pool size = (2,2) |
| Conv 1D | Number of Filters = 32,  Filter Size = (5x5),  Stride = (1,1),  Padding = Valid,  Regularizer = L2 (0.002),  Activation Function = ReLU | Number of Filters = 20,  Filter Size = (5x5),  Stride = (1,1),  Padding = Valid,  Regularizer = L2 (0.002),  Activation Function = ReLU |
| Dropout | Dropout ratio = 0.5 | Dropout ratio = 0.5 |
| Max-pooling | pool size = (2,2) | pool size = (2,2) |
| Dense | Number of neurons = 512,  Activation Function = ReLU, | Number of neurons = 256,  Activation Function = ReLU, |
| Output dense | Number of neurons = 7,  Activation Function = Softmax | Number of neurons = 1,  Activation Function = Sigmoid |
| LOSS function | Categorical cross entropy + Regularization term | Binary cross entropy + Regularization term |
| Optimizer | ADAM | ADAM |
| Batch Size | 1000 | 20 |
| Epoch number | 100 | 20 |
| Learning Rate | 0.001 | 0.001 |
| Performance Metrics | Accuracy, Precision, Sensitivity, Specificity, F1 Score, Loss | Accuracy, Precision, Sensitivity, Specificity, F1 Score, Loss |
