# Supplementary material for: Artificial intelligence based diagnosis of sulcus: assesment of videostroboscopy via deep learning
Source: Eur Arch Otorhinolaryngol. 2024 Jul 13;281(11):6083–91. doi: 10.1007/s00405-024-08801-y (PMC11512876; doi:10.1007/s00405-024-08801-y)
Supplement: Supplementary file 2 — Supplementary Material 2 [file 405_2024_8801_MOESM2_ESM.docx]

**ARTIFICIAL INTELLIGENCE BASED DIAGNOSIS OF SULCUS: ASSESMENT OF VIDEOSTROBOSCOPY VIA DEEP LEARNING**

Ömer Tarık Kavak^1^, Şevket Gündüz^2^, Cabir Vural^3^, Necati Enver^1*^

1:Marmara University Faculty of Medicine, Pendik Training and Research Hospital, Department of Otorhinolaryngology, İstanbul, Turkey

2:VRLab Academy

3:Marmara University Faculty of Engineering, Electrical and Electronics Engineering, İstanbul, Turkey

^*^Corresponding Author: Ömer Tarık Kavak M.D.

Adress: Fevzi Çakmak, Muhsin Yazıcıoğlu Street, 34899, İstanbul, Turkey

[omrkavak11@gmail.com](mailto:omrkavak11@gmail.com) , orcid ID: 0000-0003-2603-3866

+90 531 915 01 31

Author: Şevket Gündüz, Assistant Professor of Physics

Adress: 32 Willoughby Rd, Harringay Ladder, London N8 0JG, United Kingdom

[sevketgunduz@gmail.com](mailto:sevketgunduz@gmail.com.r) , orcid ID: 0000-0002-1847-949X

+90 530 883 49 83

Author: Cabir Vural, Professor of Electrical and Electronics Engineering

Adress: Başıbüyük, RTE Campus, 34854, İstanbul, Turkey

[cabir.vural@marmara.edu.tr](mailto:cabir.vural@marmara.edu.tr) , orcid ID: 0000-0003-2603-3866

+90 555 733 39 87

Author: Necati Enver M.D., Associate Professor of Otorhinolaryngology, FEBORL-HNS

Adress: Fevzi Çakmak, Muhsin Yazıcıoğlu Street, 34899, İstanbul, Turkey

[necatienver@gmail.com](mailto:necatienver@gmail.com) , orcid ID: 0000-0002-3161-8810

+90 532 338 20 88

**METHODS**

**Model Construction**

The study involved development of two classifiers. Based on the existing literature, it is evident that sulcus represents the most commonly undiagnosed benign VF lesion, and it may appear normal during indirect laryngoscopy. Considering this, a binary classifier was developed with the purpose of distinguishing an image corresponding to a patient diagnosed with sulcus from an image corresponding to a healthy subject. Subsequently, a multi-class classifier was developed to classify a given image into one of seven distinct categories, including healthy, polyp, nodule, papilloma, sulcus, cyst, and pseudocyst.

The study presented an 8-layer CNN based model for both classifiers. The overall architecture of the model, illustrated in Supplementary Figure 1, includes two convolutional layers, each accompanied by dropout and max-pooling layers. Convolutional layers extract class specific features from a given frame. After the convolutional layers, a Fully Connected Layer (FCL) consisting of two dense layers is incorporated. FCL has two outputs in the case of binary classifier while it has seven outputs for multi-class classification.

2D-Convolution

$$X(F^{\left( i \right)})$$

FCL

$$Y(X^{(i)})$$

$$\hat{Y}^{(i)}$$

Dropout

2D-Convolution

Dropout

Maxpooling

2D-Image $\mathbf{F}^{\left( i \right)}$

Maxpooling

Flatten

Dense Layer

Dense Layer

**Supplementary Figure 1.** Block diagram of the proposed CNN model. F is the input image, $X$ stands for the extracted feature, $Y$ is the ouput of the fully connected layer and $\hat{Y}$is the predicted class.

The model was constructed using Keras with Tensor Flow backend, (version 2.8.0 for both). The implementation was done in Python, (version 3.9.10 for Windows). Implementation was seamlessly integrated into the customized software for smooth execution.

**Model Training, Validation and Testing.**

The Adaptive Momentum (ADAM) algorithm was employed to iteratively optimize the model parameters using a minibatch containing $m$ examples from the training set. The cost function encompassing two loss functions, is computed over the entire minibatch. The first loss function is defined as the *binary cross-entropy* function for binary, and *categorical cross-entropy* (equivalently softmax) function for multi-classification. The second loss function is the $l_{2}$-norm of all parameters. $l_{2}$-norm serves as a regularization function to avoid overfitting. In order to find the best model, a systematic approach was employed in which the model hyperparameters were iteratively modified. After each adjustment, the model was retrained and validated to evaluate its performance. This process continued until the most desirable outcome was achieved. The number of epochs served as the termination criterion for each training iteration. Supporting Table 1 shows the optimal values of the underlined hyperparameters. Figure 3 illustrates accuracy and loss curves with respect to epoch number during training and validation processes. These curves were achieved with the optimal hyperparameters of the CNN-based models.
